# The TGF-**β**/HDAC7 axis suppresses TCA cycle metabolism in renal cancer

**DOI:** 10.1172/jci.insight.148438

**Published:** 2021-11-22

**Authors:** Hyeyoung Nam, Anirban Kundu, Suman Karki, Garrett J. Brinkley, Darshan S. Chandrashekar, Richard L. Kirkman, Juan Liu, Maria V. Liberti, Jason W. Locasale, Tanecia Mitchell, Sooryanarayana Varambally, Sunil Sudarshan

**Affiliations:** 1 Department of Urology and; 2 Department of Pathology, University of Alabama at Birmingham (UAB), Birmingham, Alabama, USA.; 3 Department of Pharmacology and Cancer Biology, Duke University, Durham, North Carolina, USA.; 4 O’Neal Comprehensive Cancer Center, UAB, Birmingham, Alabama, USA.; 5 Birmingham Veterans Affairs Medical Center, Birmingham, Alabama, USA.

**Keywords:** Cell Biology, Cancer, Mitochondria, Molecular biology

## Abstract

Mounting evidence points to alterations in mitochondrial metabolism in renal cell carcinoma (RCC). However, the mechanisms that regulate the TCA cycle in RCC remain uncharacterized. Here, we demonstrate that loss of TCA cycle enzyme expression is retained in RCC metastatic tissues. Moreover, proteomic analysis demonstrates that reduced TCA cycle enzyme expression is far more pronounced in RCC relative to other tumor types. Loss of TCA cycle enzyme expression is correlated with reduced expression of the transcription factor PGC-1**α**, which is also lost in RCC tissues. PGC-1**α** reexpression in RCC cells restores the expression of TCA cycle enzymes in vitro and in vivo and leads to enhanced glucose carbon incorporation into TCA cycle intermediates. Mechanistically, TGF-**β** signaling, in concert with histone deacetylase 7 (HDAC7), suppresses TCA cycle enzyme expression. Our studies show that pharmacologic inhibition of TGF-**β** restores the expression of TCA cycle enzymes and suppresses tumor growth in an orthotopic model of RCC. Taken together, this investigation reveals a potentially novel role for the TGF-**β**/HDAC7 axis in global suppression of TCA cycle enzymes in RCC and provides insight into the molecular basis of altered mitochondrial metabolism in this malignancy.

## Graphical abstract

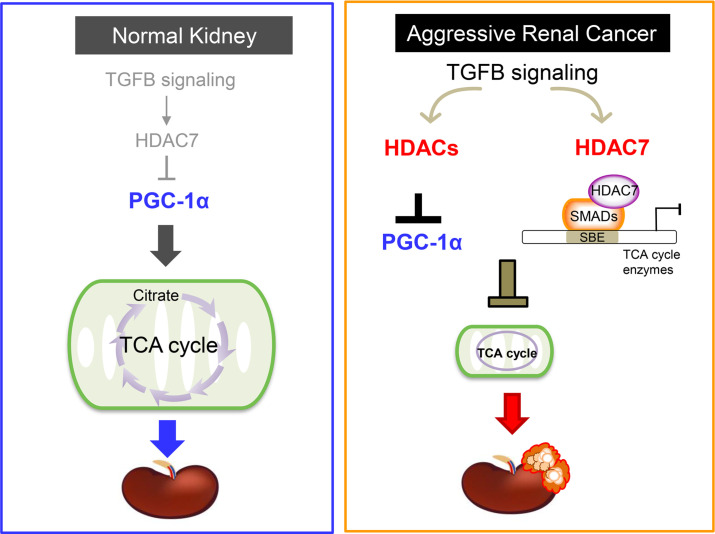

## Introduction

Clear cell renal cell carcinoma (ccRCC) is the most common histologic subtype of kidney cancer. Approximately 30%–40% of patients with ccRCC present with metastases at initial diagnosis (1, 2). Individuals with organ-confined RCC tumors are considered to have an excellent prognosis with treatment. In contrast, patients with advanced disease have poor survival rates. Thus, a better understanding of the factors leading to tumor progression in RCC and the development of novel therapeutic strategies are of potential significance. ccRCC is known to have striking metabolic features. The most well characterized is increased expression of glycolytic genes that results from loss/mutation of *VHL*, a common tumor initiating event. Loss of the E3 ubiquitin ligase activity of *VHL* results in stabilization of the hypoxia inducible factors (HIFs) and subsequent upregulation of hypoxia-responsive genes (3–5). Glycolytic enzymes are known HIF transcriptional targets in cancer (6, 7).

While enhanced glycolysis is a shared feature of many caner types, the expression of enzymes involved in mitochondrial metabolism is more heterogeneous. Notably, ccRCC is among the tumor types with the most prominent decrease in the mRNA expression of TCA cycle enzymes (8). In agreement, we previously noted reduced expression of fumarate hydratase (FH) protein in ccRCC (9). Moreover, a recent stable isotope labeling study in ccRCC patients demonstrated reduced incorporation of glucose-derived carbons into TCA cycle metabolites (10). These data are among the most compelling to demonstrate reduced TCA cycle metabolism in kidney cancer. The decreased mRNA expression of TCA cycle enzymes in kidney tumor likely has biological relevance. A major conclusion of the The Cancer Genome Atlas (TCGA) analysis of kidney cancer was a metabolic shift in aggressive tumors marked by the downregulation of genes encoding enzymes of the TCA cycle, including *FH*; aconitase 2 (*ACO2*); succinate-CoA ligase, α subunit (*SUCLG1*); and oxoglutarate dehydrogenase (*OGDH*) (11). Despite these findings, the molecular basis by which the TCA cycle is altered in ccRCC remains poorly understood. As a result, the implications of these alterations in RCC tumor biology remain unknown.

We recently reported an integrative analysis on RCC tumor progression that included normal kidney, primary tumors as well as metastatic tissues (12). This analysis demonstrated that loss of mRNA expression of *PPARGC1A*, which encodes for the transcription factor PGC-1α, as among the most suppressed genes. PGC-1α was originally identified as a transcriptional coactivator involved in mitochondrial function and thermogenesis in brown fat (13). *PPARGC1A* is known to be expressed in metabolically active tissues such as the kidney. Recently, we identified a role for PGC-1α in suppressing both the expression of collagen genes and tumor progression in an orthotopic model of RCC (14). Prior studies have demonstrated that the transcriptional regulation of collagen gene expression is mediated by TGF-β (15). Although TGF-β has been implicated in invasive behaviors via its promotion of the epithelial-to-mesenchymal transition (EMT) in many tumors, its role in mitochondrial TCA cycle metabolism is undefined. These observations led us to consider the interrelationship between TGF-β and PGC-1α and the relevance of this axis to metabolic reprogramming in the context of RCC.

Here, we demonstrate that global repression of TCA cycle enzymes is a unique feature of RCC that is also found in RCC metastatic deposits. Mechanistically, TGF-β and histone deacetylase 7 (HDAC7) cooperate to repress PGC-1α and TCA cycle enzyme expression. Moreover, pharmacologic inhibition of TGF-β can restore TCA cycle enzyme expression in vivo. Overall, our findings provide potentially novel insight into the epigenetic basis of altered mitochondrial metabolism in RCC. Moreover, they are among the first data to our knowledge to demonstrate that mitochondrial aspects of classic Warburg metabolism are pharmacologically targetable in RCC.

## Results

### The expression of TCA cycle enzymes is decreased in ccRCC.

**Figure 1:**
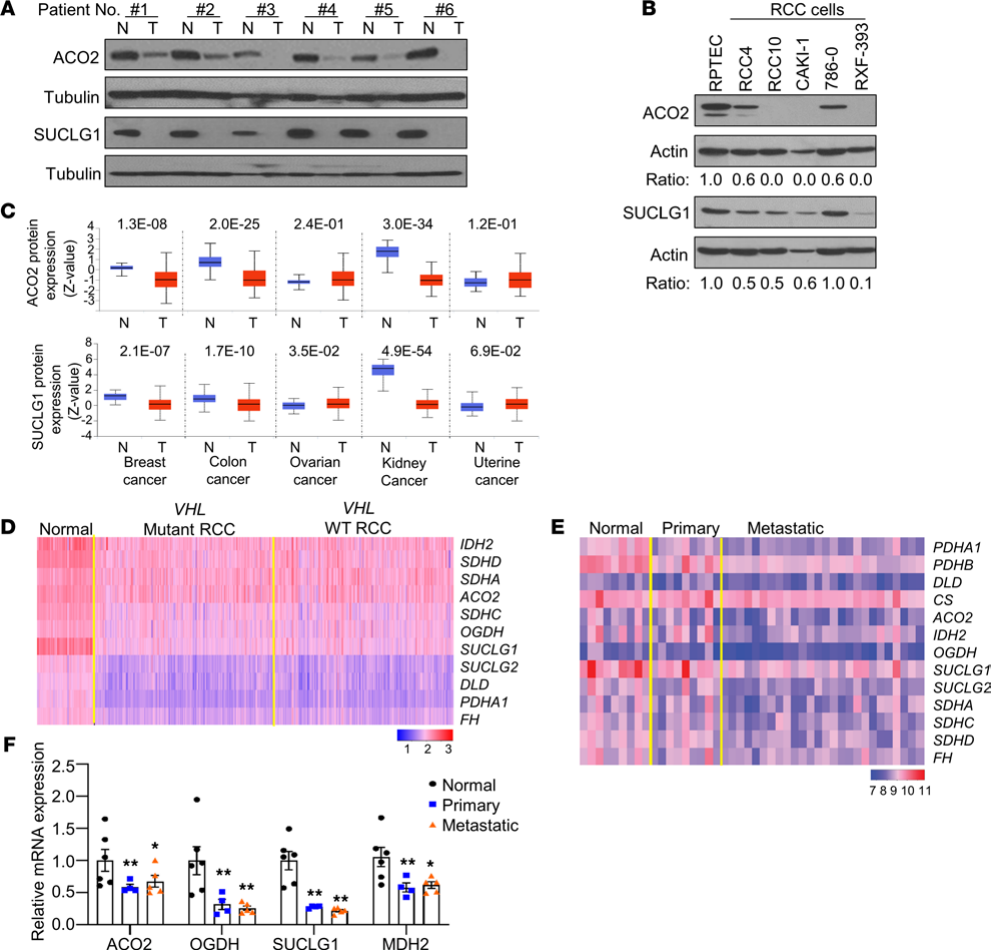
Suppression of TCA cycle enzymes in ccRCC. (**A**) Immunoblot analysis of ACO2 and SUCLG1 in patient-matched normal kidney (N) and tumor (T) (*n* = 6). (**B**) Western blot analysis of ACO2 and SUCLG1 in a panel of RCC cell lines relative to RPTEC primary renal proximal tubule epithelial cells. The band intensities for ACO2 and SUCLG1 protein levels were quantified with reference to actin control bands using ImageJ program (NIH). (**C**) Protein expression of ACO2 and SUCLG1 across cancer subtypes from the CPTAC cohorts. (**D**) Heatmap representing expression patterns of TCA cycle enzymes in normal kidney (*n* = 72), *VHL* mutant RCC (*n* = 224), and *VHL* WT RCC (*n* = 225). Data were extracted from TCGA KIRC data set. The heatmap shows the log_10_-transformed TPM values for each gene. (**E**) Heatmap representing expression patterns of TCA cycle enzymes using the Illumina Human HT-12 v4 bead array in the 3-patient groups (normal, *n* = 9; primary, *n* = 9; and metastasis, *n* = 26). Colors in the heatmap represent log-transformed quantile normalized expression values. (**F**) Relative mRNA expression of TCA cycle enzymes in a separate cohort of patient-matched samples. Transcript levels were normalized to those of *TBP* (*n* = 4–6). Asterisks indicate significant differences compared with normal kidney (*P < 0.05, ** *P* < 0.01, 1-way ANOVA with Tukey’s multiple-comparison test).

Prior TCGA analyses demonstrated reduced mRNA expression of genes encoding TCA cycle enzymes. Apart from our previous study on FH (9), these data have not been validated at the protein level. We first examined primary ccRCC specimens and patient-matched adjacent normal kidney and found reduced protein levels of ACO2 and SUCLG1 in RCC (Figure 1A). In addition, we characterized the relative expression of TCA cycle enzymes in a panel of RCC cell lines. In general, RCC cell lines had decreased protein levels of ACO2 and SUCLG1 relative to RPTEC renal proximal tubule epithelial cells (RPTEC) (Figure 1B). *VHL* alterations are the most common tumor-initiating event in ccRCC. An established sequelae of *VHL* loss leads to the stabilization of HIFs, and this has been linked with alterations in mitochondrial metabolism (16). Both *VHL* mutant (RCC4 and RCC10) and *VHL* WT (CAKI-1 and RXF-393) lines demonstrated reduced TCA cycle enzyme protein expression. Interestingly, *VHL* mutant 786-0 cells, which express HIF-2α but not HIF-1α, retained the expression of SUCLG1 protein. We analyzed publicly available proteomics data released from TCGA (CPTAC) using the UALCAN (http://ualcan.path.uab.edu/) analysis portal (17). These data validated our findings of reduced protein expression of TCA cycle enzymes in ccRCC relative to normal kidney. Analysis of proteomics data from other tumor types demonstrates that reduced expression of TCA cycle enzymes is far more pronounced in ccRCC with greater statistical significance relative to other tumor types (Figure 1C and Supplemental Figure 1; supplemental material available online with this article; https://doi.org/10.1172/jci.insight.148438DS1). Whereas breast and colon cancers demonstrated slightly reduced expression, no changes were noted in ovarian and uterine cancers. We also assessed the relative mRNA expression of TCA cycle enzymes by *VHL* status in ccRCC from the TCGA data set. Consistent with our findings in cell lines, we found that both *VHL* WT and mutant tumors demonstrated reduced mRNA expression of TCA cycle enzymes relative to normal kidney (Figure 1D). We next considered whether these changes were maintained in RCC metastatic tissues, as TCGA only analyzed primary tumors. We recently reported the gene expression landscape of ccRCC progression, which encompassed transcriptomic analysis of normal kidney (*n* = 9), primary RCC (*n* = 9), and metastatic RCC tissue deposits (*n* = 26) (12). Notably, we found that the mRNA expression of TCA cycle enzymes is reduced in metastatic tissues, indicating that the pronounced shift in TCA cycle metabolism is retained with tumor progression (Figure 1E). Since these samples were not patient matched, we examined the expression of TCA cycle enzymes in a separate cohort, which included patient-matched samples of normal kidney, primary tumors, and metastatic tissues. This analysis confirmed that the reduced mRNA expression of TCA cycle enzymes (i.e., *ACO2*, *OGDH*, *SUCLG1*, and *MDH2*) in primary tumors was retained in metastatic tissues (Figure 1F).

### The transcriptional landscape of ccRCC reveals a positive correlation between TCA cycle enzymes and PPARGC1A.

**Figure 2:**
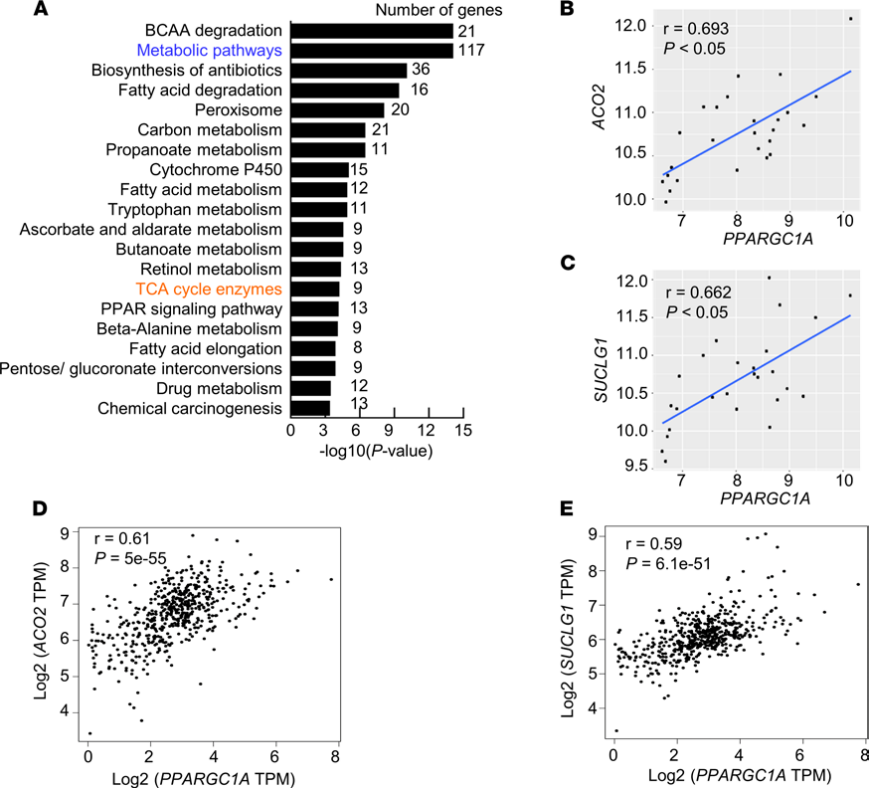
*PPARGC1A* expression is positively correlated with the expression of TCA cycle enzymes in ccRCC. (**A**) KEGG pathway analysis representing that the pathways are positively correlated with *PPARGC1A* in metastatic RCC samples (*n* = 26). (**B** and **C**) Positive correlation between *PPARGC1A* and TCA cycle enzymes in metastatic tumor samples determined by Pearson’s correlation (*n* = 26). (**D** and **E**) Results of correlation analysis between *PPARGC1A* and TCA cycle enzymes (*ACO2* and *SUCLG1*) in TCGA KIRC data set for renal tumors. Data extracted using GEPIA web server.

We next wanted to gain insight into the mechanism that drives the suppression of TCA cycle enzyme in RCC. We recently reported that *PPARGC1A* expression is progressively silenced with RCC tumor progression (14). Restoration of PGC-1α suppresses in vivo tumor progression in an orthotopic model of RCC (14). PGC-1α is known to have a role in mitochondrial metabolism. We first performed correlation analysis of genes positively correlated with *PPARGC1A* in metastatic RCC tissues. KEGG analysis demonstrated that metabolic pathways including TCA cycle enzymes were among the most enriched pathways (Figure 2A). Several genes of the TCA cycle enzyme demonstrated a statistically significant positive correlation with *PPARGC1A* including *ACO2*, *SUCLG1*, succinate dehydrogenase A (*SDHA*), *SDHB*, succinate-CoA ligase ADP-forming subunit β (*SUCLA2*), and dihydrolipoamide S-acetyltransferase (*DLAT*) (Figure 2, B and C, and Supplemental Table 1). Furthermore, a positive correlation between *PPARGC1A* and several TCA cycle enzyme genes was also observed upon analysis of TCGA data (Figure 2, D and E).

### PPARGC1A reexpression restores the expression of TCA cycle enzymes and mitochondrial function.

**Figure 3:**
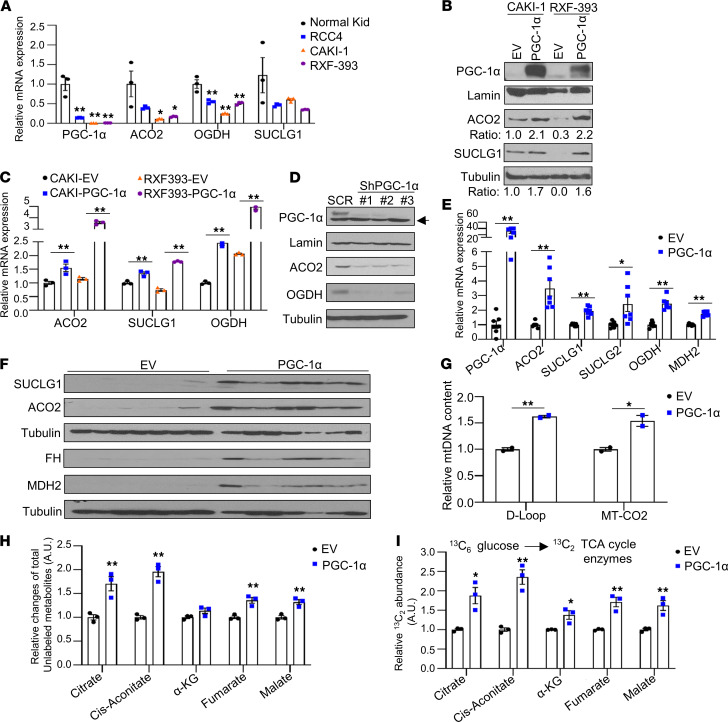
PGC-1α reexpression upregulates the expression of TCA cycle enzymes and glucose flux to TCA cycle enzymes. (**A**) Relative mRNA expression of the indicated genes in a panel of RCC cells relative to normal kidney (*n* = 3). Transcript levels were normalized to those of *TBP* (*P < 0.05, **P < 0.01, 1-way ANOVA with Tukey’s multiple-comparison test). (**B**) Western blot analysis of the indicated proteins in RCC cells stably expressing EV or PGC-1α. Relative intensities for target protein were quantified using ImageJ software. (**C**) mRNA expression of TCA cycle enzymes from RCC cells transduced either EV or PGC-1α (*n* = 3). Data represent mean ± SEM (**P < 0.01, 2-tailed Student’s *t* test). Data are representative of 3 independent experiments. (**D**) Western blot analysis of the indicated proteins in 769-P cells stably expressing shRNA control (SCR) or 3 independent PGC-1α shRNA constructs. Arrow represents nonspecific band. (**E** and **F**) SN12PM6-1 cells stably expressing EV or PGC-1α were orthotopically implanted into the left kidney of SCID mice. At 6 weeks from tumor challenge, kidney tissues were harvested and analyzed for the expression of mRNA (**E**) and protein for the indicated genes (**F**) (*n* = 7). (**G**) CAKI-1 cells stably expressing EV or PGC-1α were measured for the amount of mtDNA D-Loop structure and *MT-CO2* gene (*n* = 2). (**H** and **I**) CAKI-1 cells stably expressing with EV or PGC-1α were incubated with uniformly labeled [U-^13^C_6_] glucose for 24 hours. The relative levels of total unlabeled metabolites (**H**) and labeled TCA cycle intermediates (**I**) (M+2) were analyzed using LC-MS (*P < 0.05, **P < 0.01, 2-tailed Student’s *t* test).

The correlation studies led us to consider the role of PGC-1α loss on the expression of TCA cycle enzymes. Consistent with the decreased mRNA expression of *PPARGC1A*, RCC cell lines demonstrated a significant decrease in mRNA expression of TCA cycle enzymes, including *ACO2*, *OGDH*, and *SUCLG1*, relative to normal kidney (Figure 3A). PGC-1α expression was restored in CAKI-1 and RXF-393 RCC cells. PGC-1α restoration led to increased protein expression of ACO2 and SUCLG1 in RCC cells (Figure 3B). Consistent with the protein data, PGC-1α reexpression significantly increased the mRNA expression of TCA cycle enzymes in both RCC cells (Figure 3C). Similar findings were found in RCC4 cells transduced with adenovirus containing cDNA for PGC-1α (Supplemental Figure 2, A and B). Knockdown of PGC-1α using the 3 independent shRNA constructs in 769-P cells, which have a low but detectable level of PGC-1α protein (14), led to reduced protein expression of ACO2 and OGDH (Figure 3D). Moreover, these findings were relevant in vivo, as restoration of PGC-1α in SN12PM6-1 RCC cells led to increased mRNA and protein levels of TCA cycle enzymes in orthotopic xenografts (Figure 3, E and F).

We next examined the effect of PGC-1α on mitochondrial DNA (mtDNA) content. The amount of both mtDNA D-Loop structure and *MT-CO2* gene, encoding for mtDNA-encoded cytochrome C oxidase II (MT-CO2), was significantly increased in PGC-1α expressing RCC cells (Figure 3G). We next assessed the effects of PGC-1α on TCA cycle activity with liquid chromatography–mass spectrometry (LC-MS) analysis using uniformly labeled [U-^13^C_6_] glucose. First, we noted that PGC-1α increased total unlabeled pools of multiple TCA cycle metabolites including citrate, cis-aconitate, fumarate, and malate (Figure 3H). Furthermore, PGC-1α led to increased labeling of TCA cycle metabolites, indicating the enhanced contribution of glucose-derived carbons to the TCA cycle (Figure 3I). Collectively, these data indicate that PGC-1α restoration in RCC cells can promote the expression of TCA cycle enzymes, as well as increase mtDNA content and enzyme activity.

### Blockade of TGF-β signaling rescues the expression of PPARGC1A and mitochondrial function in RCC.

**Figure 4:**
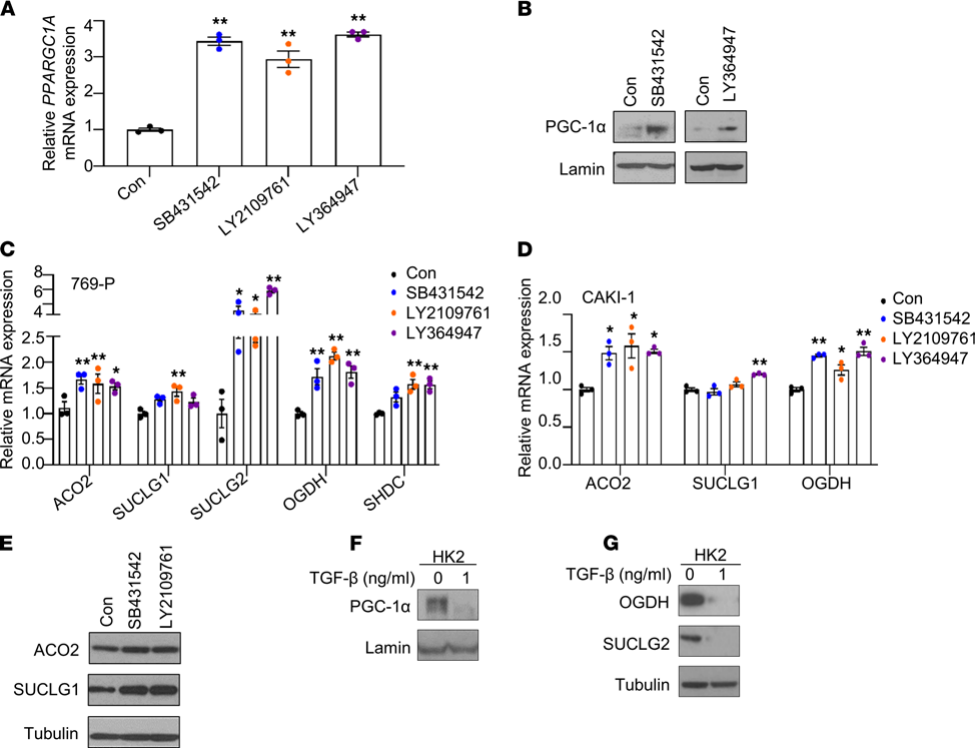
The expressions of *PPARGC1A* and TCA cycle enzymes are restored by blockade of TGF-β signaling. (**A**) Relative mRNA expression of *PPARGC1A* in CAKI-1 cells treated with either DMSO (Con) or indicated pharmacological TGF-β inhibitors (10 μM) for 48 hours (*n* = 3). (**B**) Immunoblot analysis for PGC-1α protein expression in nuclear lysate from 769-P cells treated with indicated TGF-β inhibitors for 48 hours. (**C** and **D**) Relative mRNA expression of TCA cycle enzymes in 769-P and CAKI-1 cells treated with either DMSO or indicated TGF-β inhibitors (10 μM) for 48 hours (*n* = 3) (*P < 0.05, **P < 0.01, 1-way ANOVA with Tukey’s multiple-comparison test). (**E**) Immunoblot analysis for ACO2 and SUCLG1 protein expression in cell lysates from CAKI-1 cells treated with the indicated TGF-β inhibitor for 48 hours. (**F**) Western blot analysis of PGC-1α in nuclear lysates from HK2 cells exposed to TGF-β (1 ng/mL) for 24 hours. Lamin B1 was used as a loading control. (**G**) HK2 cells were exposed to TGF-β (1 ng/mL) for 24 hours, followed by immunoblot analysis for OGDH and SUCLG2 expression. Data are representative of 2–3 independent experiments. The unedited versions of all blot images are provided in Supplemental Figure 6.

The profound role of PGC-1α on the expression of TCA cycle enzymes led us to consider the mechanism that promotes loss of PGC-1α expression in RCC. Prior studies have indicated that loss of PGC-1α is HIF dependent in RCC (18). However, our analysis of TCGA data demonstrates that TCA cycle enzyme expression is reduced irrespective of *VHL* status, indicating an alternate mechanism (Figure 1D). We recently reported that the increased mRNA expression of collagen (*COL*) family members is highly associated with RCC metastasis and that PGC-1α restoration suppresses the expression of *COLs* (14). Prior studies have indicated a role for TGF-β in promoting *COL* gene expression as part of the EMT program (15). We therefore assessed TGF-β’s role in regulating the expression of PGC-1α. Consistent with TGF-β’s role in promoting *COL* expression, we found that pharmacologic inhibition of TGF-β suppressed the mRNA expression of several *COL* genes including *COL1A1*, *COL5A1*, *COL5A2*, and *COL11A1* in CAKI-1 and RXF-393 cells (Supplemental Figure 3, A and B). Consistent with the mRNA data, inhibition of TGF-β led to decreased protein expression of COL1A1 in RCC cells (Supplemental Figure 3C). Inhibition of TGF-β signaling utilizing multiple inhibitors (SB431542, LY2109761, and LY364947) resulted in an increase of *PPARGC1A* transcript levels in RCC cells (Figure 4A and Supplemental Figure 3D). Correspondingly, inhibition of TGF-β signaling led to increased protein expression of PGC-1α in RCC cells (Figure 4B). In addition, TGF-β inhibition led to a significant increase in mRNA expression of the TCA cycle enzymes in 769-P and CAKI-1 cells (Figure 4, C and D). Increased protein levels of ACO2 and SUCLG1 were observed in CAKI-1 cells treated with TGF-β inhibitors (Figure 4E). Furthermore, HK2 renal epithelial cells treated with TGF-β resulted in a decrease in protein expression of PGC-1α (Figure 4F). In addition, the treatment of TGF-β in HK2 cells led to reduced protein expression of TCA cycle enzymes (Figure 4G).

### The effect of TGF-β inhibition on cellular bioenergetics and TCA cycle enzyme expression is abolished upon PGC-1α knockdown.

**Figure 5:**
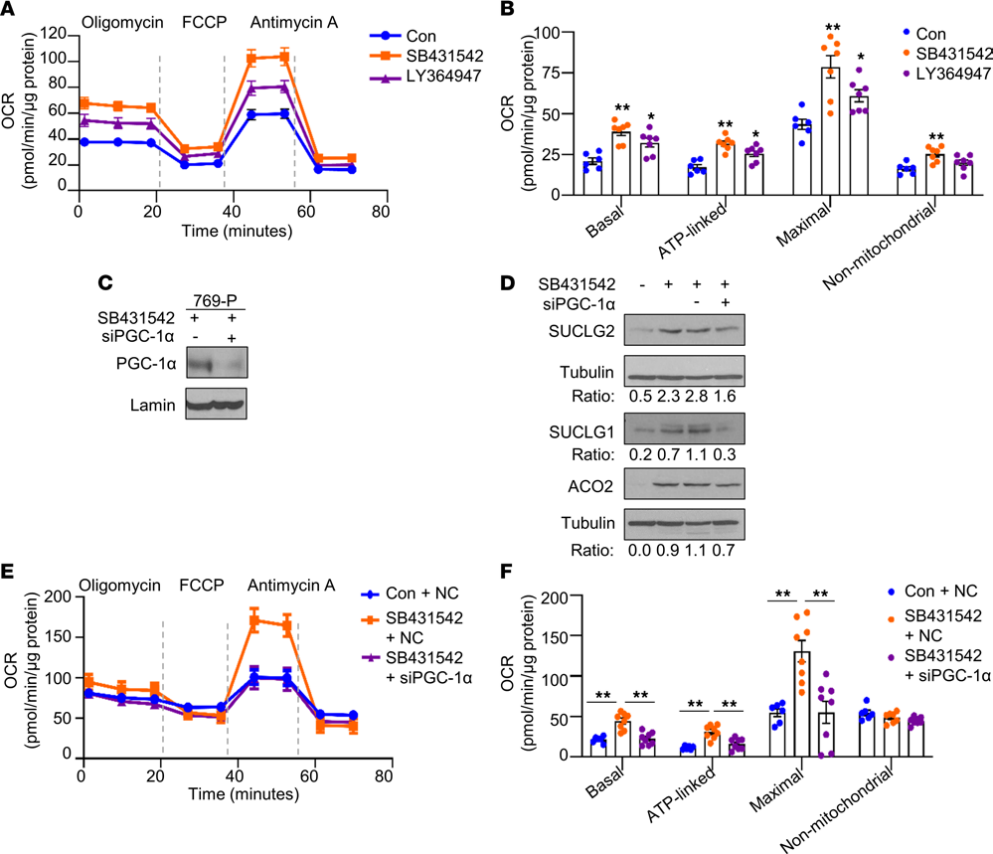
The effect of TGF-β inhibition on cellular bioenergetics and TCA cycle enzyme expression is abolished in PGC-1α–deficient cells. (**A** and **B**) Oxygen consumption rate (OCR) was analyzed in CAKI-1 cells treated with either DMSO (Con) or indicated pharmacological TGF-β inhibitor (10 μM) for 48 hours (*n* = 6~8). Oligomycin (1.5 μg/mL), FCCP (0.6 μM), and antimycin A (10 μM) were sequentially added to the cells. Representative cellular bioenergetic profiles (**A**) and individual parameters (**B**) are shown. (**C**) Immunoblot analysis for PGC-1α in nuclear lysates from 769-P cells transfected with either 50 nM negative control or PGC-1α siRNA in the presence of SB431542 (20 μM) for 48 hours. (**D**) Immunoblot analysis for TCA cycle enzymes in cell lysates from 769-P cells transfected with either NC or PGC-1α siRNA in the presence of SB431542 for 48 hours. Relative intensities for SUCLG2 and ACO2 protein expressions were quantified using ImageJ software. (**E** and **F**) OCR was analyzed in 769-P cells transfected with either 50 nM NC or PGC-1α siRNA in the absence or presence of SB431542 (20 μM) for 48 hours. OCR data are representative of 2 independent experiments, and data are means ± SEM; *n* = 6–8. Asterisks indicate differences relative to control (*P < 0.05, **P < 0.01, 1-way ANOVA with Tukey’s multiple-comparison test). The unedited versions of all blot images are provided in Supplemental Figure 6.

Based on these observations, we examined the impact of TGF-β inhibition on cellular bioenergetics. We measured the oxygen consumption rate (OCR) of RCC cells treated with either control (DMSO) or 2 pharmacological TGF-β inhibitors (Figure 5A). Notably, both TGF-β inhibitors increased cellular bioenergetics in RCC cells compared with control cells. In particular, both inhibitors significantly increased basal respiration, ATP-linked respiration (assessed following oligomycin treatment), and maximal respiration (assessed following treatment with the uncoupler FCCP) (Figure 5B). SB431542 significantly increased nonmitochondrial respiration (following antimycin A treatment), whereas LY364947 had no effect (Figure 5B). Collectively, these data demonstrate a role for TGF-β signaling in regulating both TCA cycle enzyme expression and cellular bioenergetics in RCC cells. We next assessed if restoration of TCA cycle enzymes and OCR by TGF-β inhibition is PGC-1α dependent. We first confirmed siRNA-mediated knockdown of PGC-1α expression in the presence of TGF-β inhibitor (SB431542) in 769-P cells (Figure 5C). Consistent with previous findings, SB431542 treatment in 769-P cells transfected with control siRNA led to increased protein expression of TCA cycle enzymes, including SUCLG2, SUCLG1, and ACO2 (Figure 5D). In contrast, PGC-1α knockdown dampened the induction of TCA cycle enzymes by TGF-β inhibitor SB431542 (Figure 5D). Given these findings, we further examined the role of TGF-β inhibition on cellular bioenergetics in PGC-1α–knockdown cells. 769-P cells were treated with TGF-β inhibitor following the transfection with either negative control (NC) or siRNA for PGC-1α. As shown in Figure 5, E and F, PGC-1α knockdown nullified the increase in OCR with TGF-β inhibition. Taken together, these data demonstrate that the effects of TGF-β inhibition on TCA cycle enzyme expression and cellular bioenergetics are PGC-1α dependent.

### TGF-β inhibition reverses metabolic phenotypes of RCC in vivo.

**Figure 6:**
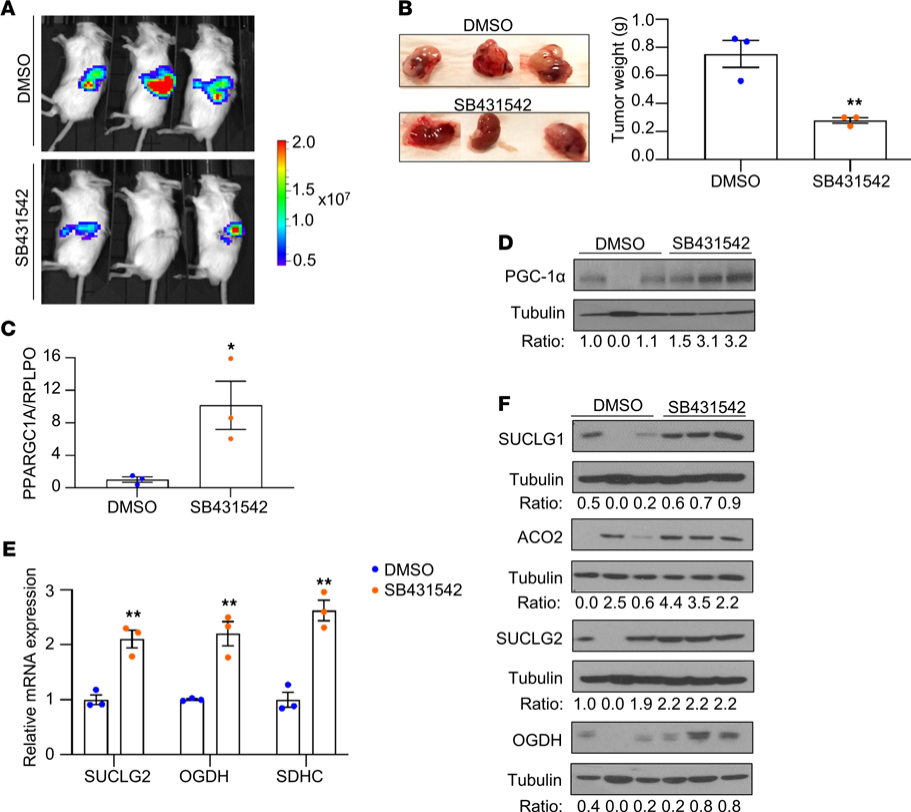
Inhibition of TGF-β signaling suppresses tumor formation and restores the expression of *PPARGC1A* and TCA cycle enzymes in vivo. (**A**) CAKI-1 luciferase-expressing cells (1.5 × 10^6^) were implanted into renal subcapsular region of SCID mice (*n* = 3). Tumor formation was confirmed 1 week after cell injection, and mice were injected i.p. with either DMSO or SB431542 three times per week for 5 weeks (10 mg/kg in 20% DMSO). The in vivo luciferase bioluminescence was taken at 5 weeks after treatment with either DMSO or SB431542. (**B**) At 5 weeks after treatment, kidney tissues were harvested and weighed. (**C**) The RNA was isolated from kidney tumors of mice treated with either DMSO or SB431542 and analyzed for mRNA expression of *PPARGC1A*. Transcript levels were normalized to those of *RPLPO* (*P < 0.05, **P < 0.01, 2-tailed Student’s *t* test). (**D**) Immunoblot analysis of PGC-1α in kidney tissues from the mice treated with either DMSO or SB431542. (**E** and **F**) Relative mRNA and protein expression of TCA cycle enzymes in kidney tissues from the mice treated with either DMSO or SB431542. Relative intensities for target protein were quantified using ImageJ software. Transcript levels were normalized to those of *RPLPO*. All data are presented as ± SEM; *n* = 3 (*P < 0.05, **P < 0.01, 2-tailed Student’s *t* test).

As noted previously, a major finding from the TCGA analysis of ccRCC was that loss of TCA cycle enzyme expression is associated with aggressive tumors. Given our in vitro findings, we next assessed if these findings are relevant in vivo to assess if mitochondrial aspects of Warburg metabolism could be reversed by pharmacologic means. CAKI-1 luciferase–expressing RCC cells were injected orthotopically into the renal subcapsular region of SCID mice. RCC tumor–bearing mice were then i.p. treated with either 20% DMSO (control) or TGF-β inhibitor SB431542 (10 mg/kg in 20% DMSO). After 5 weeks of treatment, mice treated with SB431542 demonstrated significantly lower tumor burden, as demonstrated by both bioluminescent imaging (Figure 6A) and tumor weight (Figure 6B). Analysis of tumor explants demonstrated increased expression of *PPARCG1A* mRNA and PGC-1α protein in mice treated with SB431542 (Figure 6, C and D). Moreover, TGF-β inhibitor–treated tumors demonstrated increased expression of TCA cycle enzymes at both the mRNA and protein levels (Figure 6, E and F). Collectively, these data demonstrate the role of TGF-β in regulating the TCA cycle in RCC and that this can be targeted by pharmacologic means in vivo.

### HDAC7 acts as a corepressor for TGF-β–mediated suppression of TCA cycle enzymes in RCC.

**Figure 7:**
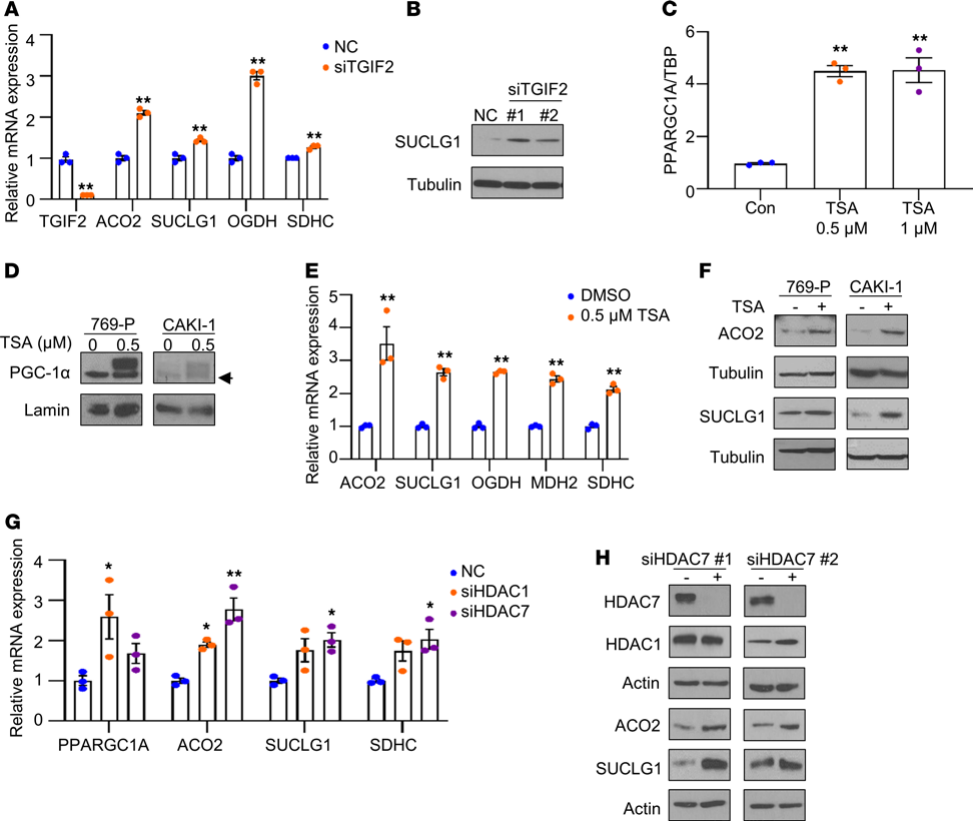
The expression of TCA cycle enzymes is restored by HDAC7 knockdown. (**A**) The mRNA expression of TCA cycle enzymes in CAKI-1 cells transfected with negative control (NC) or TGIF2 siRNA for 72 hours (*n* = 3). (**B**) SUCLG1 protein expression in CAKI-1 cells transfected with NC or 2 independent TGIF2 siRNA constructs for 72 hours. (**C**) The mRNA expression of *PPARGC1A* in CAKI-1 cells treated with Trichostatin A (TSA) for 24 hours (*n* = 3). (**D**) Immunoblot analysis for PGC-1α in nuclear lysate from 769-P and CAKI-1 cells treated with TSA for 24 hours. Arrow represents nonspecific band. (**E**) The mRNA expression of TCA cycle enzymes in CAKI-1 cells treated with TSA for 24 hours. Data are representative of 3 independent experiments and show mean ± SEM. Asterisks indicate significant differences compared with control (**P < 0.01, 2-tailed Student’s *t* test). (**F**) Immunoblot analysis for TCA cycle enzymes in cell lysate from 769-P and CAKI-1 cells treated with TSA for 24 hours. (**G**) 769-P cells were transfected with the indicated HDAC siRNA or NC for 72 hours. Relative mRNA expression was analyzed for TCA cycle enzymes (*n* = 3). Asterisks indicate significant differences compared with NC (*P < 0.05, **P < 0.01, 1-way ANOVA with Tukey’s multiple-comparison test). (**H**) Immunoblot analysis for the indicated proteins in cell lysates from 769-P cells transfected with either NC or 2 independent HDAC7 siRNA constructs for 72 hours. Date are representative of at least 2 independent experiments.

We next investigated the mechanism by which TGF-β signaling represses the expression of *PPARGC1A* and TCA cycle enzymes. TGF-β signaling regulates transcription by a complex network, including SMAD proteins. SMAD complexes can inhibit transcription through interacting with transcriptional corepressors. Three SMAD corepressors have been identified, including the homeodomain protein TG-interacting factors (TGIFs), Ski, and SnoN protein (19–21). We therefore examined genes that are negatively correlated with these corepressors in the TCGA data set on ccRCC using the Genomic regression analysis of coordinated expression (GRACE) and Gene expression profiling interactive analysis (GEPIA) (22). Intriguingly, this analysis identified that *TGIF2* is inversely correlated with the expression of genes involved in the TCA cycle enzymes (Supplemental Table 2 and Supplemental Figure 4A). In fact, the TCA cycle was the top-ranked gene set. Based on these findings, we next performed loss-of-function studies via siRNA-mediated knockdown of *TGIF2* in CAKI-1 cells. Knockdown of *TGIF2* led to significantly increased mRNA levels of TCA cycle enzymes, including *ACO2*, *SUCLG1*, *OGDH*, and *SDHC* (Figure 7A). Correspondingly, the protein levels of SUCLG1 were upregulated by knockdown of *TGIF2* (Figure 7B). TGIF2 had been previously shown to be a transcriptional repressor by interacting with HDACs including HDAC1 (21, 23). We therefore assessed whether HDACs could contribute to silencing the expression of *PPARGC1A* and/or TCA cycle enzyme genes. We initially investigated the effects of the pan-HDAC inhibitor trichostatin A (TSA) on the expression of *PPARGC1A* in RCC cells. We found a significant increase in mRNA and protein expression of PGC-1α in RCC cells following treatment with TSA (Figure 7, C and D). Furthermore, TSA treatment increased the mRNA expression of TCA cycle enzymes in CAKI-1 cells (Figure 7E). In concert, TSA treatment resulted in the increased protein expression of ACO2 and SUCLG1 in 769-P and CAKI-1 cells (Figure 7F). We therefore assessed whether HDACs inversely correlated with TCA cycle enzymes (Supplemental Table 3). We found that *HDAC1* and *HDAC7* are negatively correlated with the mRNA expression of TCA cycle enzymes in the TCGA data set (Supplemental Figure 4, B–D). We thus knocked down both HDAC1 and HDAC7. We confirmed target gene knockdown via quantitative PCR (qPCR) (Supplemental Figure 5A). We found that HDAC1 knockdown significantly induced the mRNA expression of *PPARGC1A* in 769-P cells (Figure 7G). HDAC7 knockdown had a modest effect on *PPARGC1A* mRNA. However, HDAC7 knockdown led to a prominent increase in the mRNA levels of *ACO2*, *SUCLG1*, and *SDHC* (Figure 7G). In agreement with the mRNA data, HDAC7 knockdown demonstrated increased ACO2 and SUCLG1 protein expression in RCC (Figure 7H). Collectively, these data indicate a role for histone deacetylation in suppressing TCA cycle enzyme expression with a potentially novel role for HDAC7 in silencing the TCA cycle enzyme expression.

**Figure 8:**
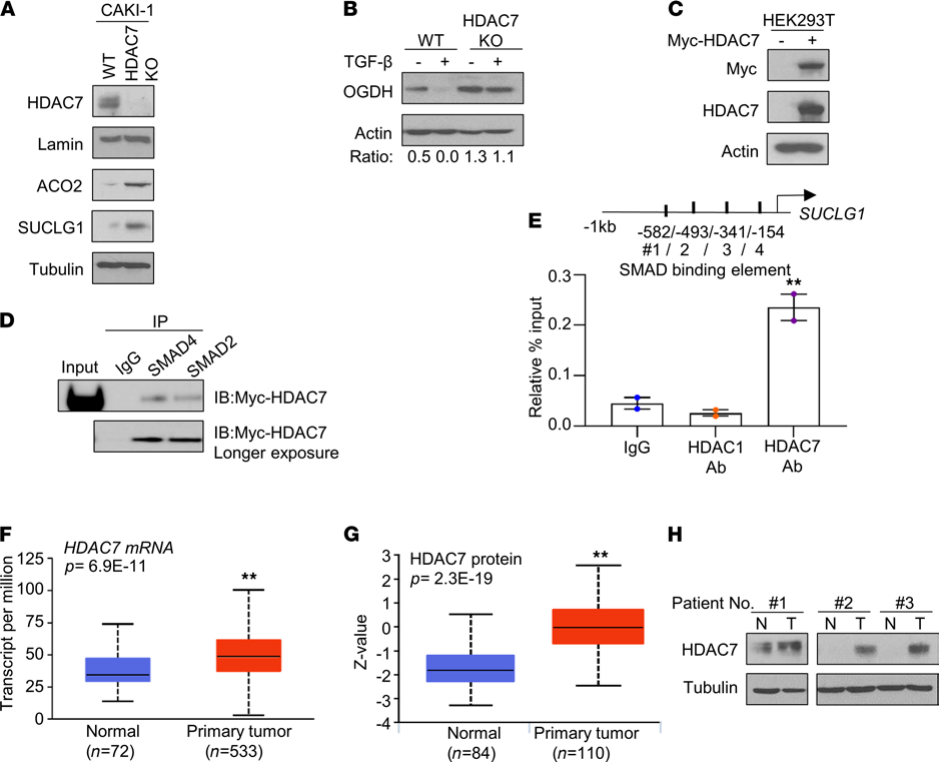
HDAC7/SMAD complex represses the TCA cycle enzyme expression. (**A**) Western blot analysis of the indicated protein levels in WT CAKI-1 (WT) and HDAC7 CRISPR–KO CAKI-1 (HDAC7-KO). (**B**) WT CAKI-1 and HDAC7-KO cells were treated with or without TGF-β (1 ng/mL) for 24 hours, followed by the immunoblot analysis for OGDH. (**C**) Immunoblot analysis for Myc and HDAC7 in HEK293T cells transfected with EV or Myc-tagged HDAC7 for 48 hours. (**D**) HEK293T cells transfecting with Myc-tagged HDAC7 were immunoprecipitated using either anti-SMAD4, anti-SMAD2, or control IgG. IP samples with individual SMAD antibody were followed by immunoblotting (IB) for Myc-tagged HDAC7. IgG pulldown is included as a control. (**E**) ChIP-qPCR was performed on CAKI-1 cells with mouse IgG, anti-HDAC1, and anti-HDAC7. The enriched DNA was quantified by qPCR with primer sets targeting the potential SMAD binding sites upstream of the *SUCLG1* transcription start site. Enrichment was calculated with the percent input method (*n* = 2/group, 3 independent experiments). Asterisks indicate significant differences compared with IgG control. (**F**) RNA-Seq analysis for *HDAC7* mRNA expression in renal tumors from the TCGA data set using UALCAN analysis. (**G**) Protein expression of HDAC7 in normal kidney and primary tumor from the CPTAC data using UALCAN analysis. (**H**) Immunoblot analysis of HDAC7 in patient-matched normal kidney (N) and tumor (T). **P < 0.01, 1-way ANOVA with Tukey’s multiple-comparison test.

We next considered the interaction between TGF-β and HDAC7 in regulating TCA cycle enzyme expression. We generated HDAC7-KO CAKI-1 cells using the CRISPR/Cas9 system. HDAC7-KO cells had undetectable protein expression of HDAC7 compared with control CAKI-1 cells (Figure 8A). In concert with our HDAC7 siRNA studies, HDAC7-KO cells had increased expression of ACO2 and SUCLG1 proteins compared with WT CAKI-1 cells (Figure 8A). Since TGF-β treatment significantly reduced TCA cycle enzyme levels in HK2 renal epithelial cells (Figure 4G), we assessed the effects of TGF-β in WT and HDAC7-KO CAKI-1 cells. TGF-β treatment significantly reduced OGDH protein levels in WT CAKI-1 cells (Figure 8B, compare lanes 1 and 2). In contrast, TGF-β had a minimal effect on OGDH protein levels in HDAC7-KO cells (Figure 8B, compare lanes 3 and 4). Prior studies have demonstrated that the SMAD proteins are downstream effectors of TGF-β signaling (24, 25). Moreover, the repressive effects on gene expression by SMADs can be mediated through physical interactions with HDACs (21, 26). To evaluate the interaction of SMADs with HDAC7, we expressed Myc-tagged HDAC7 in HEK293T cells (Figure 8C). Endogenous SMADs were immunoprecipitated and assessed for HDAC7. Immunoprecipitation with either anti-SMAD4 or anti-SMAD2 demonstrated pulldown of Myc-HDAC7, whereas control IgG pulldown did not pull down Myc-HDAC7 (Figure 8D). Immunoblotting of immunoprecipitates demonstrates successful pulldown of SMAD4 and SMAD2 (Supplemental Figure 5B). Based on these findings, we investigated potential SMAD binding sites within 1 kb of the *SUCLG1* transcription start site using the Eukaryotic Promoter Database (https://epd.epfl.ch/EPDnew_database.php) (27). We thus assessed HDAC1/7 binding to this SMAD binding site. Whereas no significant enrichment of HDAC1 was found, marked enrichment of HDAC7 was observed at putative SMAD binding sites relative to IgG control (Figure 8E and Supplemental Figure 5C). These findings indicate that the HDAC7/SMADs axis directly suppresses the expression of TCA cycle enzymes. Next, we assessed the expression of HDAC7 in ccRCC in TCGA data. We observed that HDAC7 expression was significantly increased in ccRCC relative to normal kidney at the mRNA and protein levels (Figure 8, F and G). The increased protein expression of HDAC7 in ccRCC was validated by immunoblotting patient-matched samples (Figure 8H). Collectively, these data support a novel role for HDAC7 in the silencing of TCA cycle enzymes in ccRCC.

## Discussion

The molecular basis by which tumor cells remodel their metabolism remains an area of intense investigation. Current understanding of metabolic remodeling in renal cancers has mainly focused on the HIF transcription factors (28). ccRCC is associated with inactivation of the *VHL* gene due to genetic or epigenetic alterations. *VHL* inactivation results in stabilization of HIFs and subsequent upregulation of hypoxia-responsive genes that are involved in metabolic reprogramming, including increased expression of glycolysis enzymes. Although the role of the *VHL*/*HIF* pathway is well described in RCC tumor initiation, inactivation of *VHL* alone may not be sufficient to drive ccRCC tumorigenesis (29). Furthermore, prior studies demonstrate that HIF-1α expression is often lost in ccRCC and that it actually has tumor-suppressive effects in the context of kidney cancer (28).

Despite the emphasis on increased expression of glycolytic enzymes in tumor metabolism, several lines of evidence support that alterations in mitochondrial metabolism are observed in renal cancer. For instance, prior studies have established decreased mitochondrial respiratory chain proteins in RCC and that respiratory complex chain activity is inversely correlated with prognosis (30, 31). Loss of PGC-1α has been linked to these phenotypes (18). In addition, germline loss-of-function mutations of the TCA cycle enzyme genes, including *FH*, *SDHB*, *SDHC*, and *SDHD*, have been identified in renal cancers that appear to have an increased risk for more aggressive tumors (32–34). Notably, one of the major conclusions of the TCGA data analysis of ccRCC was that loss of TCA cycle enzyme expression is associated with poorer patient outcomes (35). However, the precise mechanisms underlying this relationship have not been reported. At the same time, the global suppression of the TCA cycle enzymes suggests the possibility of a coordinated epigenetic program.

PGC-1α has been implicated in promoting tumor growth in breast cancer, pancreas cancer, and melanoma (36–38). Alternatively, PGC-1α appears to suppress metastasis in prostate cancer and in a subset of melanomas (39, 40). This inconsistency could be due to tissue-specific metabolic pathways that maintain tumor growth. We recently reported a role for PGC-1α in the suppression of collagen expression. Notably, several collagens are highly expressed in metastatic RCC (14). Given that TGF-β signaling is known to promote collagen gene expression, we investigated the impact of TGF-β in RCC metabolism and tumor progression. TGF-β is a multifunctional extracellular cytokine that regulates cell growth, differentiation, migration, and adhesion (41, 42). TGF-β can inhibit cell proliferation and is considered tumor suppressive in early stages of tumorigenesis. In contrast, TGF-β can promote tumor progression through promoting EMT (15, 43). TGF-β signaling has been linked with metabolic reprogramming in cancer cells. For instance, increased expression or activity of glycolytic enzymes during TGF-β–induced EMT has been demonstrated in multiple cancer cells (44–46). Although TGF-β signaling stimulates glycolytic phenotypes during EMT process, the effects of TGF-β on the TCA cycle enzymes in cancer remain largely unknown. Therefore, our findings add potentially novel insight into metabolic remodeling in RCC tumors. These are the first data to our knowledge to demonstrate an inhibitory effect of TGF-β on the gene expression of TCA cycle enzymes. Prior studies have implicated the impact of TGF-β on reduced transcript levels of *PPARGC1A* in renal fibrosis (47). However, the detailed molecular mechanism of TGF-β–mediated transcriptional regulation of *PPARGC1A* was not fully elucidated.

TGF-β signaling mainly regulates gene expression through receptor-mediated phosphorylation of SMAD proteins. Activated SMAD proteins translocate into the nucleus, where they bind to DNA in the regulatory region of target genes. The affinity of SMADs for DNA is weak. Thus, SMADs often cooperate with additional transcription factors in the regulation of TGF-β–responsive genes (48). Our data implicate a role for TGIF2 in regulating the TCA cycle. Our data provide compelling evidence that inhibition of TGF-β leads to increased TCA cycle enzyme expression in renal tumors. Our studies are among the first to our knowledge to demonstrate that mitochondrial aspects of the Warburg effect can be pharmacologically reversed in vivo.

Here, we also report that TGF-β works in concert with HDAC7 to suppress the expression of TCA cycle enzymes in RCC. Our data reveal that HDAC7, through interaction with SMADs, can suppress genes that are involved in mitochondrial metabolism. Moreover, our studies indicate that HDACs can suppress mitochondrial metabolism at multiple levels, including direct effects at genes encoding TCA cycle enzymes. Our findings suggest that HDACs could be targeted to alter tumor metabolism. As supportive evidence, recent studies in glioma cells indicate that HDAC inhibitors can activate mitochondrial metabolism (49). Future studies will focus on the role of mitochondrial metabolism in tumor biology, as well as a response to therapies including immune checkpoint blockade.

In summary, our findings provide insight into the molecular mechanisms driving metabolic reprogramming in renal cancer. Our studies demonstrate that TGF-β signaling represses *PPARGC1A* and TCA cycle enzymes and demonstrate a role for HDAC7/SMADs in the suppression of mitochondrial metabolism. Hence, these findings highlight an intriguing possibility that this axis could be targeted by epigenetic-based therapies that are currently in use or in clinical trials.

## Methods

### Cell culture.

RCC cell lines (RCC10, CAKI-1, 769-P, and 786-0) were purchased from the ATCC, except for RCC4 (provided by P. Ratcliffe, University of Oxford, Oxford, United Kingdom), and RXF-393 (NCI). All RCC cell lines were maintained as described previously (14). HK2 renal epithelial cells were purchased from ATCC. Primary RPTEC were acquired from Lonza and grown in renal epithelial cell growth basal medium supplemented with 10% FBS (Atlanta Biologicals) and penicillin streptomycin (100 U/mL; Corning) in 5% CO_2_ at 37°C. Cells were used within 10 passages of the initial stock and periodically screened for mycoplasma contamination.

### TGF-β inhibitor treatment.

RCC cells were grown in serum-free medium supplemented with 0.1% BSA (Roche) and 2 mM glutamine (MilliporeSigma) for 24 hours prior to treatment with either TGF-β (1 ng/mL, Calbiochem) or 10 µM TGF-β inhibitors (SB431542, Sigma-Aldrich; LY2109761 and LY364947, Selleckchem) for the indicated times. HK2 cells were cultured with 3% FBS prior to exposure with serum-free medium supplemented with 0.1% BSA (Roche) and 2 mM glutamine for 24 hours, followed by treatment with TGF-β (1 ng/mL).

### siRNA transfection.

RCC cells were transfected with 25 nM of a NC siRNA or indicated HDAC siRNA using Lipofectamine RNAiMAX reagent (Invitrogen) for 72 hours. To knock down *PPARGC1A* expression, RCC cells were transfected with 50 nM of either NC or siPGC-1α for 48 hours.

### Plasmid and virus infections.

Human *PPARGC1A* cDNA was obtained from GeneCopoeia. Lentiviral shRNA constructs for *PPARGC1A* were purchased from MilliporeSigma. Lentiviral particles were generated by cotransfecting HEK293T cells with packaging plasmids using the calcium phosphate method. The detailed methods were described previously (14). For adenoviral studies, RCC cells were transduced with either GFP or *PPARGC1A* (Vector BioLabs).

### Generation of HDAC7-KO cell lines.

To generate human HDAC7-KO in CAKI-1 cells, CRISPR targets were chosen in the coding region of exon 2 using the Massachusetts Institute of Technology CRISPR design tool (https://zlab.bio/guide-design-resources). CRISPR guide RNAs (gRNA) were subcloned into lentiCRISPR v2 (Addgene plasmid no. 52961) using BsmBI sites. gRNA sequences were (forward) 5′-CACCGCTCGGGCATCGGCGTGTCCA-3′ and (reverse) 5′-AAACTGGACACGCCGATGCCCGAGC-3′. Lentiviral particles were generated by transfecting HEK293T cells with lentiCRISPR v2 expressing gRNA for HDAC7 using the calcium phosphate method.

### Cellular bioenergetics.

Cellular bioenergetics was determined using the Seahorse XFe96 Analyzer (Agilent Technologies). RCC cells were treated with DMSO (MilliporeSigma) or TGF-β inhibitors for 24 hours prior to being plated in a Seahorse XF96 plate (25 × 10^3^ cells per well; *n* = 8/group). Cells rested overnight before measuring the OCR the following day. Cells were then washed with extracellular flux media and allowed to equilibrate for 1 hour prior to being exposed to the mitochondrial stress test. To demonstrate the effect of TGF-β inhibition on the cellular bioenergetics in PGC-1α–deficient cells, 769-P cells were treated with DMSO or 20 μM SB431542 following the transfection with either 50 nM NC or siPGC-1α using Lipofectamine RNAiMAX reagent for 24 hours prior to being plated in a Seahorse XF96 plate (35 × 10 ^3^ cells per well; *n* = 8/group). Replated cells were retreated with DMSO or 20 μM SB431542 with either 50 nM NC or siPGC-1α, as indicated.

### Patient samples and gene expression profiling.

Patient samples for gene expression profiling (normal, *n* = 9; primary, *n* = 9; metastasis, *n* = 26) were not patient matched and are previously described (12). A separate cohort of patient-matched samples (*n* = 6/group) for the validation of gene expression profiling was obtained from UAB Hospital and has been described previously (12).

### qPCR.

Total RNA from human and mouse kidney samples was harvested using the RNeasy Mini Kit (Qiagen). Total RNA isolation from cultured cells was extracted using Trizol reagent (Ambion) according to the manufacturer’s instructions. cDNA was generated using a High-Capacity cDNA Reverse Transcription Kit (Applied Biosystems). qPCR was performed using the indicated Taqman primers in QuantStudio 6K Flex Real-Time PCR System (Applied Biosystem) (Supplemental Table 4A). The mRNA expression of the target gene was normalized to either ribosomal protein (*RPLPO*) or TATA binding protein (*TBP*). The normalized Ct value was quantified using the ΔΔCt analysis.

### ChIP-qPCR.

The potential SMAD binding sites near the *SUCLG1* transcriptional start site were identified using the Eukaryotic Promoter Database (27). The ChIP experiment was conducted using the EZ-Magna ChIP Chromatin IP A/G kit (MilliporeSigma) according to the manufacturer’s instructions. After pull-down with either mouse IgG or HDAC1/7 antibody, input DNA and immunoprecipitated DNA were purified (Zymo Research). The target DNA enrichment was calculated based on the percent input method. Primer sequences for ChIP assay are described in Supplemental Table 4B.

### Measurement of mtDNA content.

DNA from RCC cells was extracted with the QIAamp DNA mini kit (Qiagen). mtDNA content was analyzed by measuring the relative levels of mtDNA D-Loop and mtDNA-encoded *MT-CO2* by qPCR. Genomic DNA–encoded β-actin was used as a normalizer. The list of primers used for this study is presented in Supplemental Table 5A.

### Immunoblotting analysis.

RCC cells were lysed with ice-cold RIPA buffer containing 1× protease inhibitor (Halt protease and phosphatase inhibitor cocktail, Thermo Fisher Scientific). Human and mouse kidney samples were homogenized with microbeads (Bioexpress) containing 1× SDS lysis buffer. Preparation of samples and Western blot analysis were described previously (12, 14). Antibodies used in this study are described in Supplemental Table 5B. The unedited versions of all blot images are provided in Supplemental Figure 6.

### Co-IP.

Human HDAC7 cDNA was cloned into pCMV-Myc (N-terminus) plasmid vector (Clontech) and verified by Sanger sequencing. HEK293T cells were transfected with Myc-tagged HDAC7 using Fugene 6 (Promega). After 48 hours, cells were harvested with lysis buffer containing 20 mM Tris-HCl, 150 mM NaCl, 0.3% Nonidet P-40, and 10% glycerol at pH 7.5 with protease/phosphatase inhibitor (Thermo Fisher Scientific). The extracted cell lysates were incubated with protein A/G-agarose (Santa Cruz Biotechnology Inc.), followed by incubation with the indicated antibody for 16 hours. The detailed experimental procedures are described previously (50).

### ^13^C glucose incorporation analysis.

CAKI-1 cells were grown to approximately 80% confluence. Cells were supplemented with fresh 5.5 mM [U-^13^C_6_] glucose (Cambridge Isotope Laboratories). After 24-hour incubation, metabolites were extracted using cold 80% HPLC graded methanol. The sample was centrifuged at 20,000*g* for 10 minutes at 4°C, and the supernatant was dried under a vacuum. Pellets were reconstituted in solvent (water/methanol/acetonitrile, 2:1:1, v/v) and further analyzed by LC-MS as previously described (51).

### Orthotopic tumor challenge in vivo.

Orthotopic tumor challenges were performed using 5-week-old SCID male mice (Charles River Laboratories). Luciferase-expressing CAKI-1 cells (1.5 × 10^6^) were mixed in a 1:1 ratio with Matrigel (Corning) and injected under the left renal capsule. Bioluminescent imaging was performed after 1 week to assess for xenograft formation. For TGF-β inhibition in vivo, SB431542 (Sigma-Aldrich) was dissolved with 20% DMSO and filtered through a 0.45 μm filter (MiliporeSigma). Mice received either 20% DMSO as a control group or diluted SB431542 in 20% DMSO (10 mg/kg) 3 times per week for 5 weeks (*n* = 3/group). Tumor progression was weekly evaluated by measuring the luciferase signal with IVIS Lumina III In Vivo System (PerkinElmer).

### Statistics.

Data are represented as mean ± SEM of at least 2–3 independent experiments. The exact number of samples is described in the corresponding figure legend. Tests of statistical significance between control and experimental groups were analyzed using 2-tailed Student’s *t* test or a 1-way ANOVA. *P* values of less than 0.05 were considered statistically significant.

### Study approval.

All animal studies were conducted in accordance with the NIH guidelines and were approved by UAB IACUC. Fresh frozen normal kidney and tumor samples were obtained in accordance with a UAB IRB–approved protocol.

## Author contributions

HN and SS designed the research, analyzed the experiments, and wrote the manuscript. AK, SK, GJB, and RLK performed in vitro experiments. DSC and SV generated and analyzed the bioinformatics data. JL, MVL, and JWL conducted and analyzed the ^13^C labeling study. TM conducted the cellular bioenergetics experiments.

## Supplementary Material

Supplemental data

Supplemental table 1

Supplemental table 2

Supplemental table 3

Supplemental table 4

Supplemental table 5

## Version 1 10/05/2021

In-Press Preview

## Version 2 11/22/2021

Electronic publication
