# Supplementary material for: The TGF-β/HDAC7 axis suppresses TCA cycle metabolism in renal cancer
Source: JCI Insight. 2021 Nov 22;6(22):e148438. doi: 10.1172/jci.insight.148438 (PMC8663777; doi:10.1172/jci.insight.148438)

SUPPLEMENTAL DATA

Supplemental Figure 1

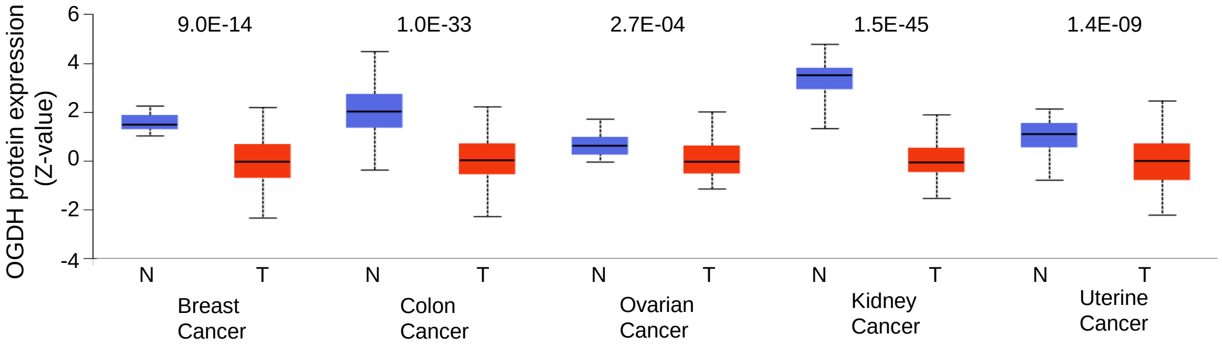

Supplemental Figure 1. The protein expression of OGDH is decreased in ccRCC. Protein expression of OGDH across pan-cancer subtypes from the CPTAC cohorts. Data were analyzed with the UALCAN analysis portal. Z-values represent standard deviations from the median across samples for the given cancer type. The levels of statistical significance are expressed as a *p*-value.

SUPPLEMENTAL DATA

Supplemental Figure 2

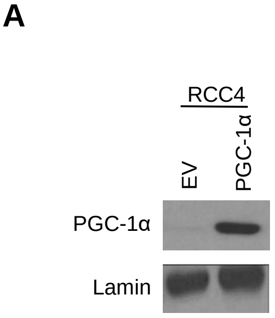

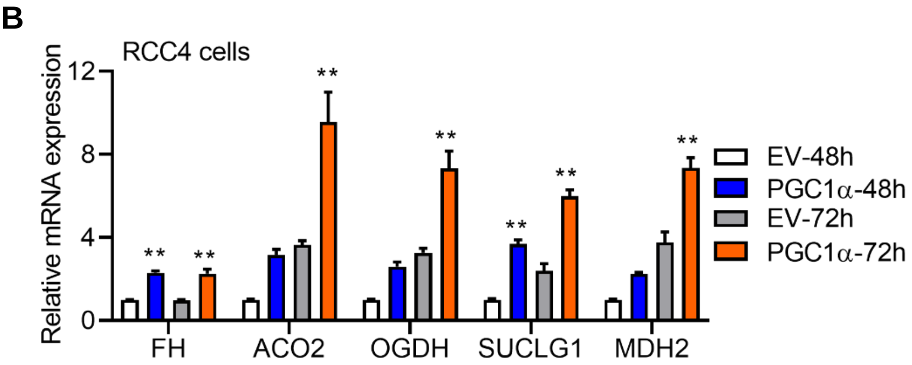

Supplemental Figure 2. *PPARGC1A* re-expression restores the mRNA expression of TCA cycle enzymes. (A) RCC4 cells transiently transduced with either Ad GFP (EV) or Ad-PGC-1α were analyzed for PGC-1α protein expression. (**B)** Representative mRNA expression of TCA cycle enzymes from RCC4 cells transiently transduced with either Ad-GFP or Ad-PGC-1α (n = 3). Data represents 3 independent experiments and error bars are SEM. Asterisks indicate differences relative to control (***P*<0.01, 2 tail student *t* test).

SUPPLEMENTAL DATA

Supplemental Figure 3

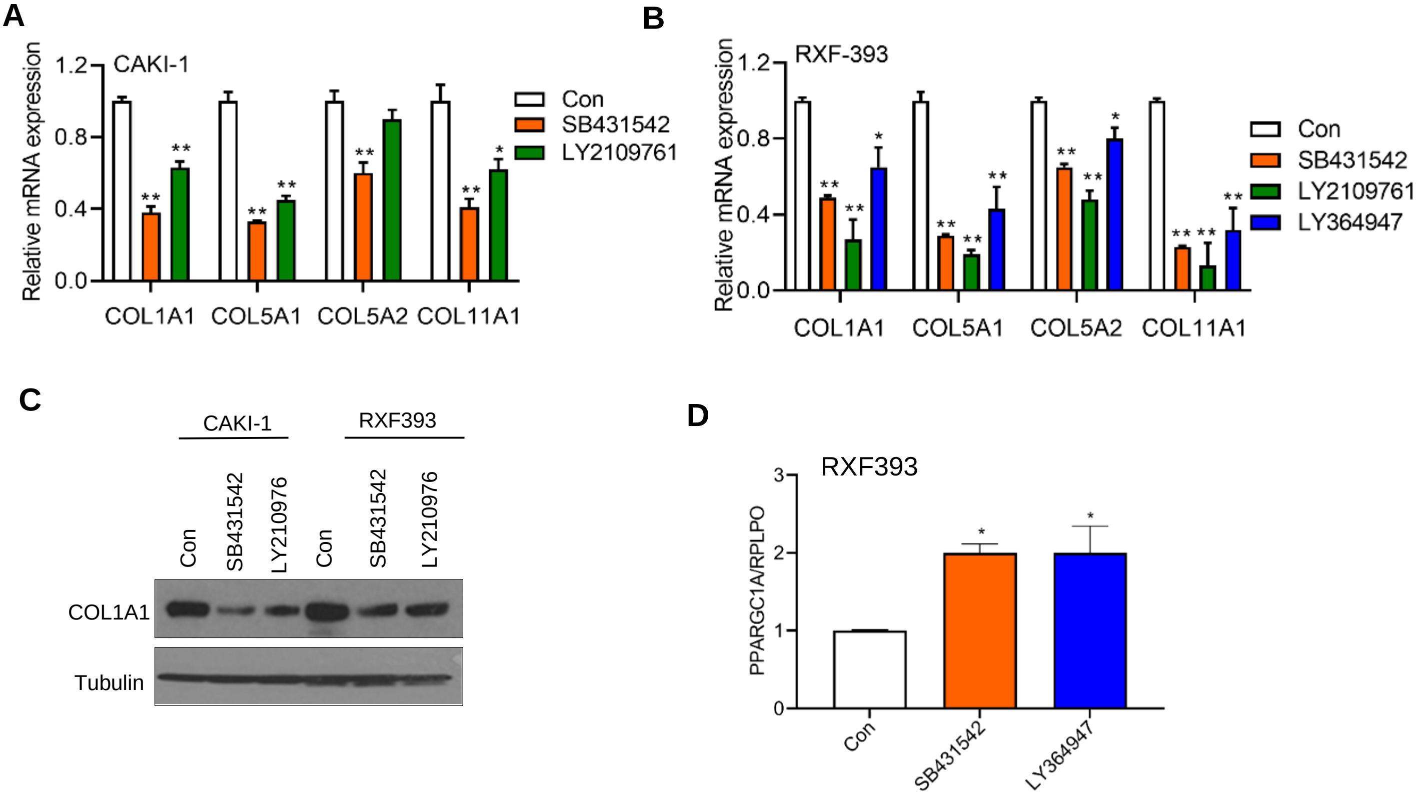

Supplemental Figure 3. Inhibition of TGF-β signaling suppresses the expression of collagen family members in RCC cells. (A) CAKI-1 cells were treated with either DMSO or indicated TGF-β inhibitors (10 μM) for 48 h (n = 3). The mRNA expression of collagen family members (*COL*) was analyzed by qRT-PCR. TBP was used for normalization. (**B**) Relative mRNA expression of *COL* in RXF-393 cells treated with either DMSO or indicated TGF-β inhibitors (10 μM) for 48 h (n = 3). (**C**) Immunoblot analysis of COL1A1 in RCC cells treated with indicated TGF-β inhibitors (10 μM) for 48 h. (**D**) Relative mRNA expression of *PPARGC1A* in RCC cells treated with either DMSO or indicated TGF-β inhibitors (10 μM) for 48 h in RXF-393 cells (n = 3). Asterisks indicate differences relative to control (*$P < 0.05$, **$P < 0.01$, One-way ANOVA with Tukey's multiple comparison test).

SUPPLEMENTAL DATA

Supplemental Figure 4

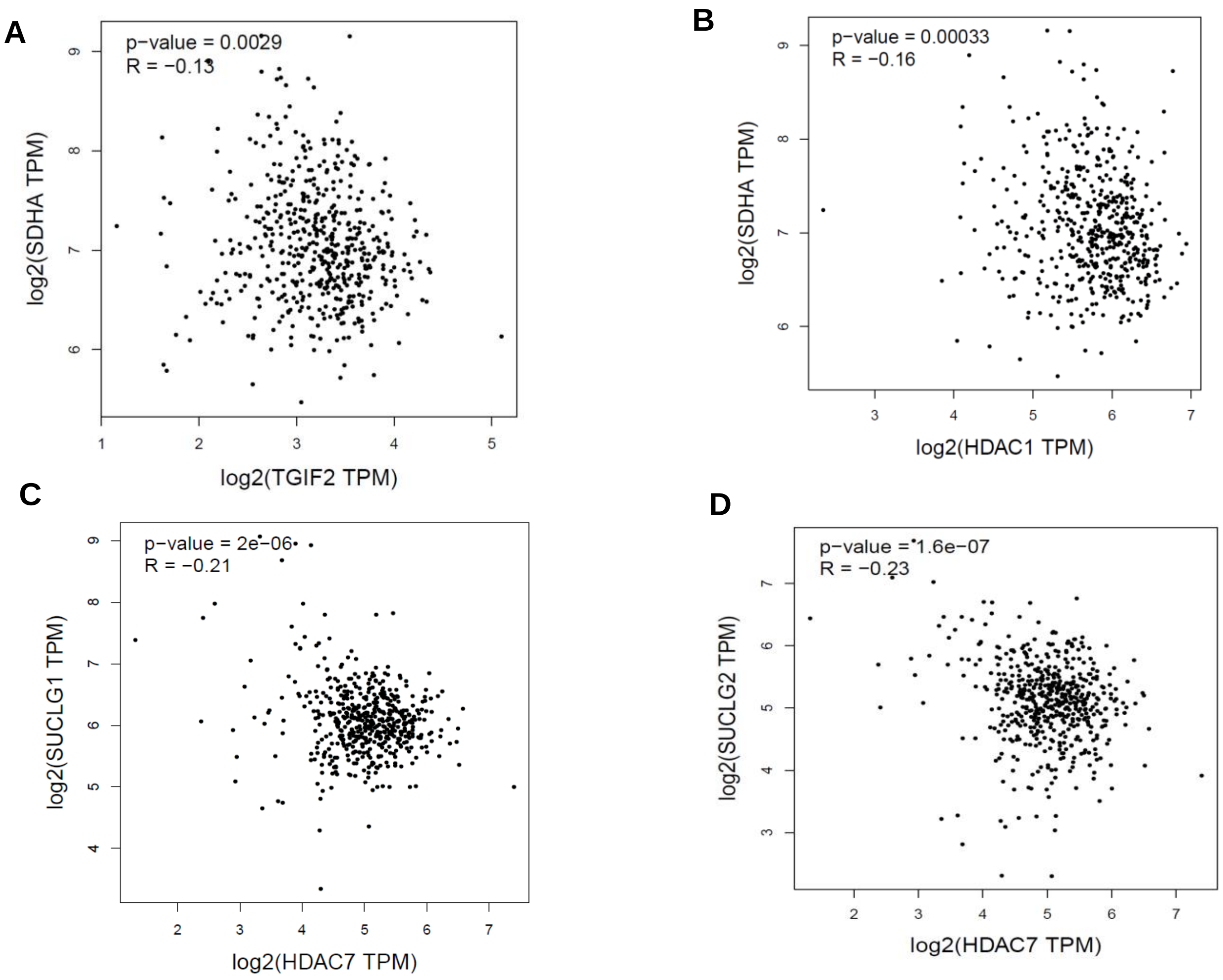

Supplemental Figure 4. *TGIF2* and *HDAC1/7* expressions are negatively correlated with the expression of TCA cycle enzymes in ccRCC. Results of correlation analysis between (**A**) *TGIF2* and *SDHA*, (**B**) *HDAC1* and *SDHA*, (**C**) *HDAC7* and *SUCLG1*, and (**D**) *HDAC7* and *SUCLG2* in RCC samples from TCGA KIRC dataset. Data extracted using GEPIA web server (http://gepia.cancer-pku.cn/detail.php?clicktag=degenes).

SUPPLEMENTAL DATA

Supplemental Figure 5

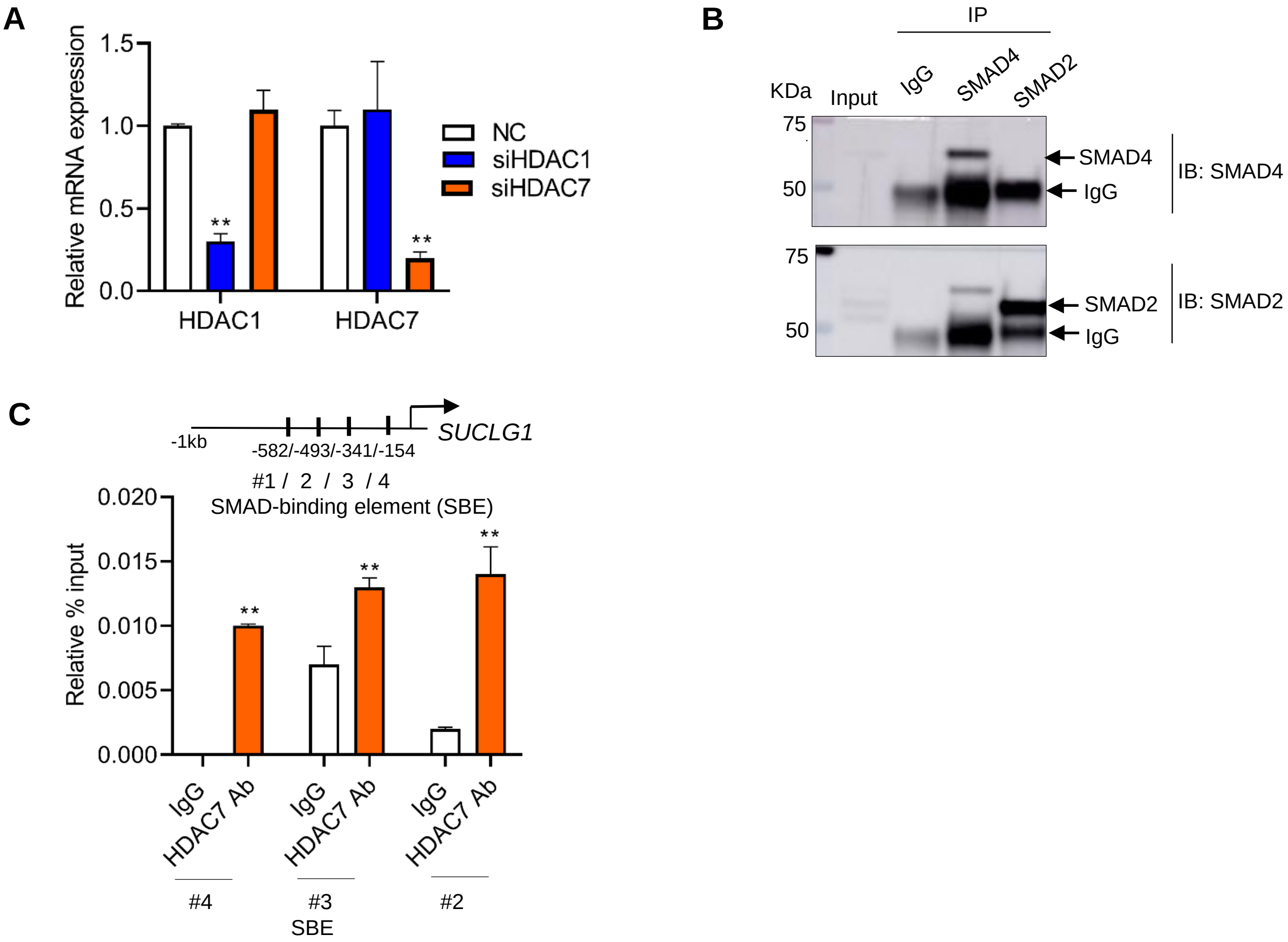

Supplemental Figure 5. HDAC7 represses the expression of TCA cycle enzymes in RCC. (**A**) Relative mRNA expression of *HDAC1* and *HDAC7* in 769-P cells transfected with 20 nM negative siRNA (NC), HDAC1, or HDAC7 siRNA for 72 h (n = 3) (***P*<0.01, 2 tail student *t* test). (**B**) HEK293T cells transfecting with Myc tagged HDAC7 were immunoprecipitated with either anti-SMAD4, anti-SMAD2, or IgG control. IP samples were immunoblotted for SMAD4 protein expression (top panel). The blot was stripped and reprobed with anti-SMAD2 antibody (bottom panel). (**C**) ChIP-qPCR was performed on CAKI-1 cells with mouse IgG and anti-HDAC7. The enriched DNA was quantified by qPCR with primer sets targeting the potential SMAD binding sites within 1.5kb upstream of the *SUCLG1* promoter. Enrichment was calculated with the percent input method (n=2, 3 independent experiments). Asterisks indicate differences relative to control (***P*<0.01, 2 tail student *t* test).

SUPPLEMENTAL DATA

Supplemental Figure 6

Figure 1A

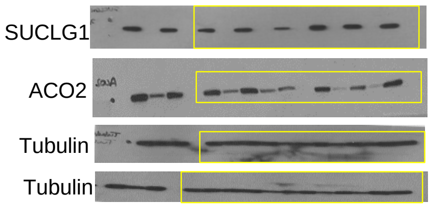

Figure 1B

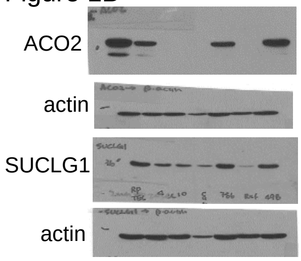

Figure 3B

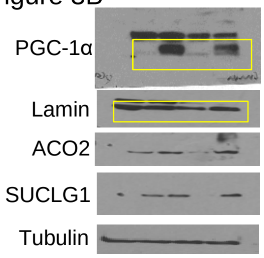

Figure 3D

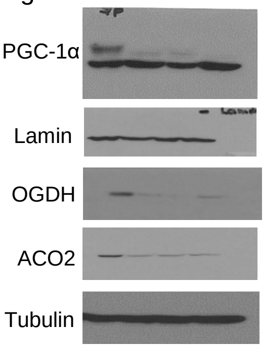

Figure 3F

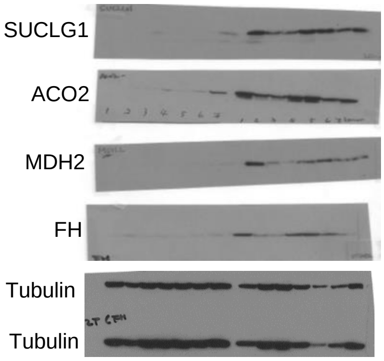

Figure 4B

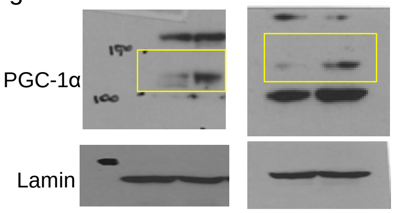

Figure 4E

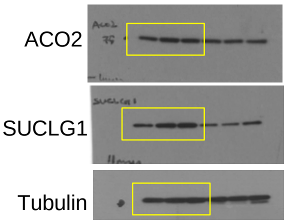

Figure 4F

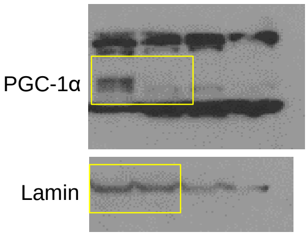

Figure 4G

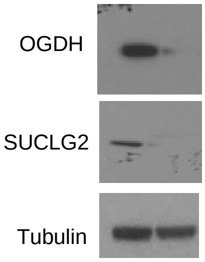

Figure 5C

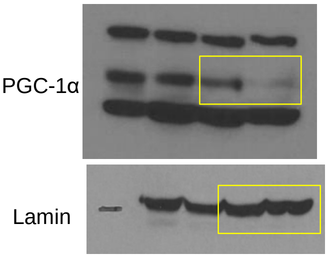

Figure 5D

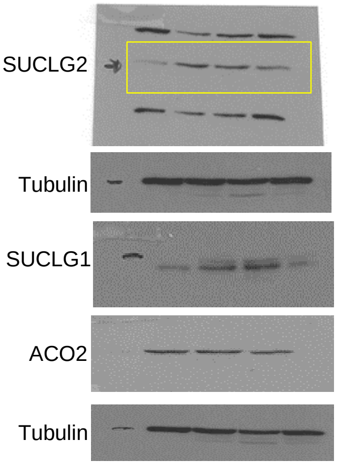

Figure 6D

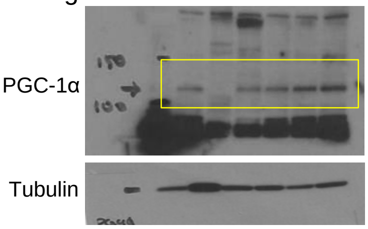

Supplemental Figure 6

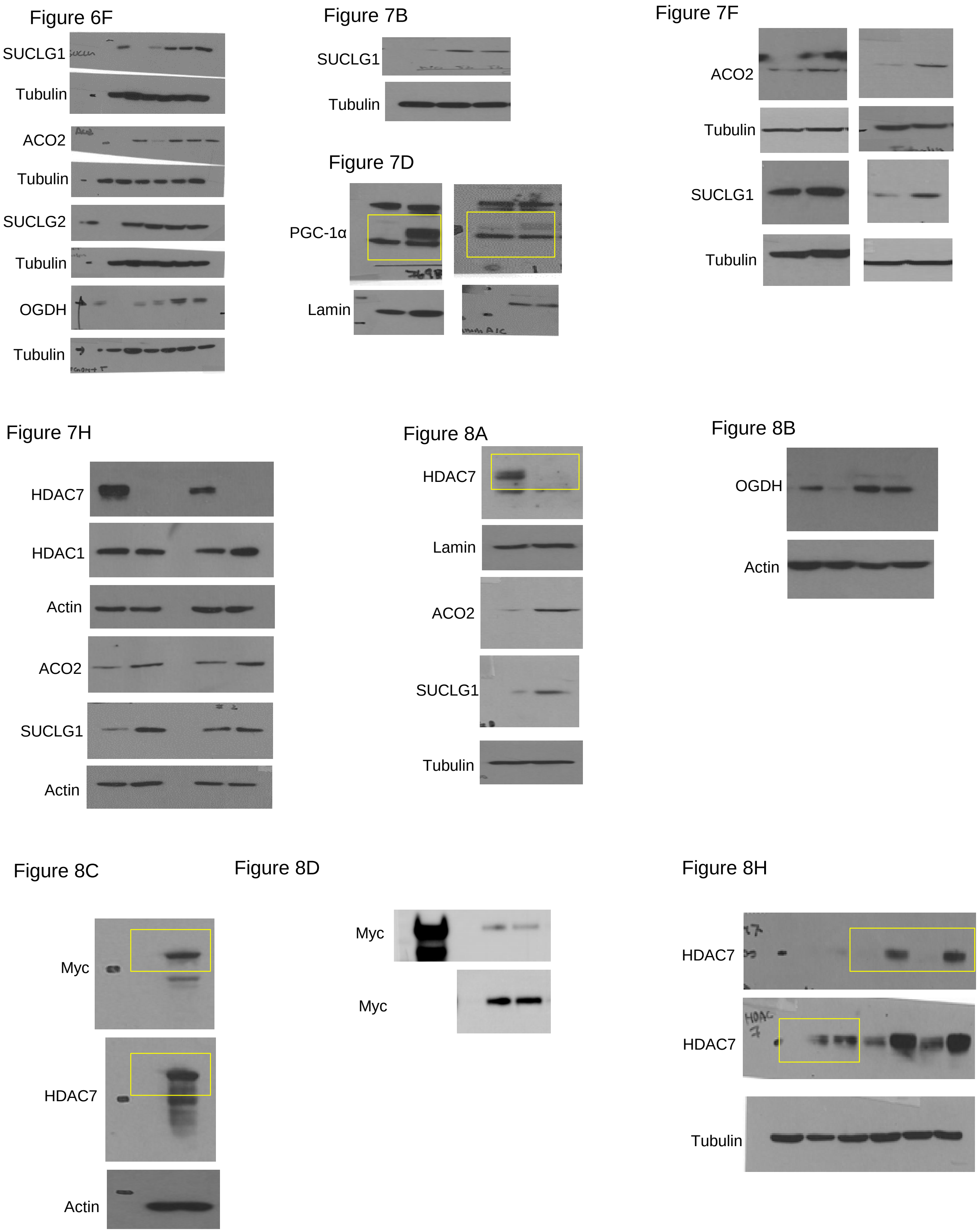

Supplement: Supplemental data [file jciinsight-6-148438-s168.pdf]
